# Technical note: durable resolution of hydrocephalus after ultrasound-guided percutaneous fenestration of giant suprasellar arachnoid cyst in a neonate

**DOI:** 10.1007/s00381-024-06560-z

**Published:** 2024-08-14

**Authors:** Michael J. Stuart, Joseph Yoon, Jane McEniery, Amelia J. Jardim, Craig Vonhoff

**Affiliations:** 1 Department of Neurosurgery, Queensland Children’s Hospital, 501 Stanley St, South Brisbane, QLD 4101 Australia; 2 College of Medicine and Dentistry, James Cook University, Townsville, QLD Australia; 3 Medical Imaging and Nuclear Medicine, Queensland Children’s Hospital, South Brisbane, QLD Australia; 4 Faculty of Medicine, University of Queensland, Brisbane, QLD Australia

**Keywords:** Arachnoid cyst, Suprasellar cyst, Minimally invasive, Ultrasound, Neonate

## Abstract

Arachnoid cysts are relatively common, but rarely require intervention. While most arachnoid cysts in typical middle or posterior cranial fossa locations are seldom symptomatic, suprasellar cysts may become symptomatic due to the potential for ventricular outflow obstruction and hydrocephalus. Typical standard of care for the treatment of these lesions is endoscopic fenestration with third ventriculostomy, or the placement of ventriculoperitoneal or cystoperitoneal shunts. The surgical and anaesthetic risks of traditional interventions may be higher in the early neonatal period, including leak of cerebrospinal fluid, infection, and premature failure of ventriculostomy or shunts. This note describes a novel bedside ultrasound-guided technique to percutaneously fenestrate large suprasellar arachnoid cysts under local anaesthesia. The technique involves insertion of a 25-g spinal needle until contact with the membrane of the arachnoid cyst medially, followed by a lateral sweeping to widely incise/fenestrate the lesion into the ventricular space under continuous ultrasound visualisation. This note describes an example case which demonstrates durable radiological and clinical improvement after 2 years of follow-up. This may represent a management option to temporise, or perhaps definitively manage suprasellar arachnoid cysts in the neonatal period.

## Introduction

Arachnoid cysts are relatively common, but rarely require intervention. While most arachnoid cysts in typical middle or posterior cranial fossa locations are seldom symptomatic, suprasellar cysts may become symptomatic due to the potential for ventricular outflow obstruction and hydrocephalus. Typical standard of care for the treatment of these lesions is endoscopic fenestration with cysto-cisternostomy, or the placement of ventriculoperitoneal or cysto-peritoneal shunts [1, 2]. While not specific to suprasellar arachnoid cysts, there are in general concerns regarding the durability of patent endoscopic third ventriculostomy in younger children [2–4]. In addition, the risks of perioperative complications may be higher in the neonatal period than in older children [3, 5].

Noting these concerns, we present the initial results of a novel ultrasound-guided percutaneous procedure which can be performed under local anaesthesia and oral sucrose ‘sedation’ which may provide a treatment option for these cysts in the early neonatal period.

## Case example

Antenatal diagnosis of a suprasellar arachnoid cyst was made at 28 weeks gestation. The cyst was measured at 2.34 × 1.8 × 2.3 cm on antenatal magnetic resonance imaging (MRI). The child was born by elective caesarian section at 39 + 1 weeks without complication. Head circumference at birth was > 99th centile. A repeat MRI was performed at 5 days of age which demonstrated a significant increase in size of the cyst to 4.7 × 6.1 × 5.9 cm, volume 72 cm^3 with progressive dilation of the lateral ventricles. Fontanelle was bulging with some splaying of sutures, but the child was appropriately alert and feeding well. No signs of Parinaud’s syndrome, bradycardias, or apnoeas were noted prior to the procedure. Pituitary function tests were normal.

## Technique

**Fig. 1:**
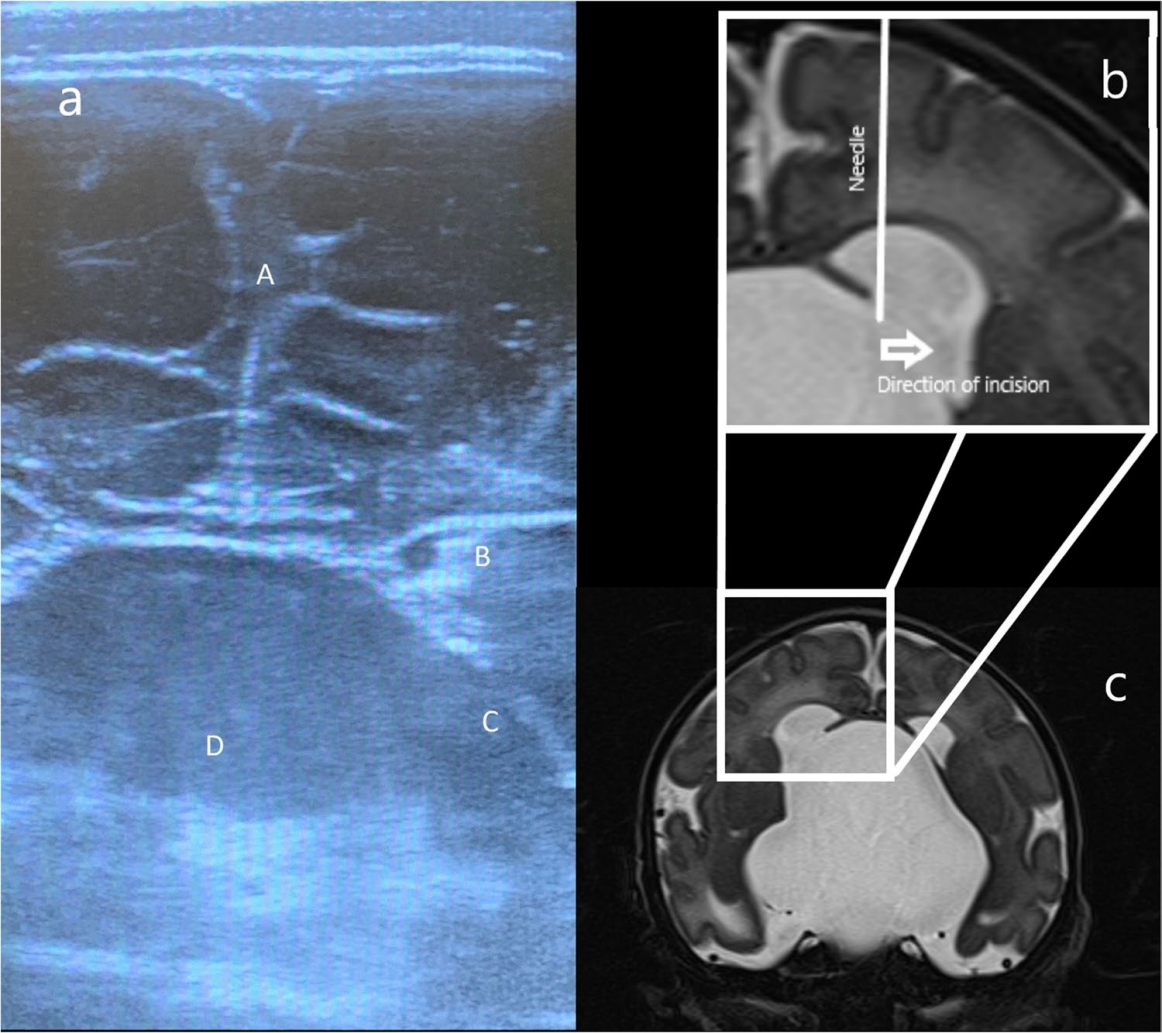
**a** Coronal ultrasound at level of foramen of Monro demonstrating needle point just traversing medial margin of arachnoid cyst as it presents into lateral ventricle. A; Falx cerebri, B; Needle tip, C; Cyst wall presenting via foramen of Monro, D; arachnoid cyst. **b** Magnified and flipped view of right lateral ventricle with needle path and trajectory of incision marked. c: coronal T2 weighted MRI scan through level of foramen of Monro

Preoperative MRI was carefully inspected to note the position and displacement of thalamostriate and septal veins. With administration of oral sucrose solution, swaddling, and infiltration of the scalp with local anaesthetic the neonate tolerated the procedure without distress. Under trans-fontanelle ultrasound guidance, a 25G spinal needle was passed through the lateral margin of the fontanelle perpendicular to the scalp surface to access the right lateral ventricle. The bevel of the needle was oriented coronally. With ongoing ultrasound guidance, the needle was advanced to just puncture and engage the medial aspect of the arachnoid cyst which presented through the foramen of Monro (Fig. 1). The needle was then swiped medial to lateral with ongoing ultrasound visualisation to ensure continued engagement of the wall of the arachnoid cyst to ensure a large fenestration/tear in the wall of the cyst. Following this manoeuvre, 20 mL of CSF was slowly aspirated over 2 min. The cyst wall was observed to collapse down into the third ventricle on ultrasound, and the fontanelle noted to be soft and sunken. The ventricles were observed with ultrasound for 5 min after withdrawal of the needle to ensure no evidence of intracranial haemorrhage was observed.

**Fig. 2:**
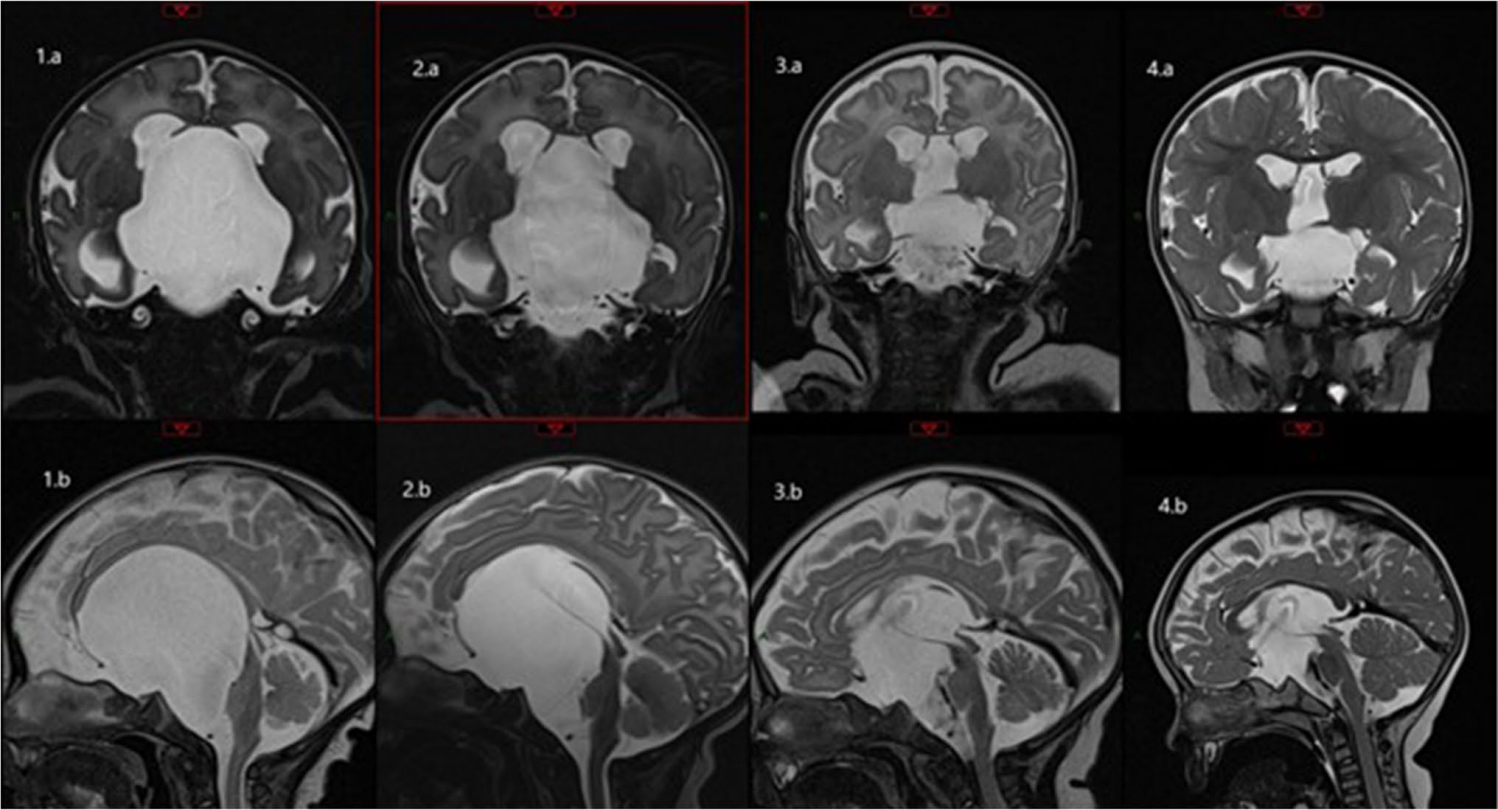
Sequential MRI demonstrating reduction in size of arachnoid cyst and ventricles. Note flow void from dorsal aspect of cyst into third ventricle. Left to right: 1. MRI at birth (a. coronal, b. sagittal), 2. MRI at D1 post procedure (a. coronal, b. sagittal), 3. MRI at 6 weeks post procedure (a. coronal, b. sagittal), 4. MRI at 14 months post procedure (a. coronal, b. sagittal)

The patient was observed in hospital for 24 h, and an MRI was repeated prior to discharge, confirming decrease in size of the cyst and ventricles and reduction in degree of local mass effect (Fig. 2). The patient was followed up with MRI at 6 weeks and 14 months post procedure which demonstrated serial decreases in size and volume of the cyst and ventricles (Table 1). The needle tract is difficult to visualise. Head circumference returned to the 97th centile and has continued to follow that growth trajectory. At time of last clinical review, 2 years post procedure, the patient was developmentally normal with no papilloedema, and no requirement for ventriculoperitoneal shunt insertion or endoscopic procedure.

**Table 1:** Serial MRI results and measurements, volumes expressed as % of postnatal, pre-procedure volume

MRI	Maximal measures	Volume of cyst (% of pre-intervention)	Evan’s index
Antenatal @28/40	2.4 × 1.8 × 2.3 cm		
Postnatal, pre-procedure	4.7 × 6.1 × 5.9 cm	72 cm^3	0.47
D1 post procedure	4.7 × 5.9 × 5.4 cm	41 cm^3 (33%)	0.44
6 weeks post procedure	4.9 × 3.4 × 4.2 cm	20 cm^3 (72%)	0.40
14 months post procedure	3.5 × 4.0 × 3.5 cm	13 cm ^3 (18%)	0.34

## Discussion

We present the first contemporary description of an ultrasound-guided, percutaneous technique for the treatment of suprasellar arachnoid cysts in neonates.

Currently standard of care consists of endoscopic fenestration of the cyst into both the ventricular space and into the prepontine cistern in a similar trajectory to a typical endoscopic third ventriculostomy. In multiple case series across several institutions the results of such techniques are reportedly very good, including in the antenatal period [1, 2, 6]. In some circumstances, alternative techniques such as ventriculoperitoneal shunt have been employed [1, 2].

Our technique may pose several advantages, specifically—this can be performed bedside under local anaesthesia and oral sucrose ‘sedation’, requires minimal equipment or consumables and results in a far smaller cortical injury from a 25-g needle relative to even the smallest current ventriculoscopes which are an order of magnitude larger in diameter. Indeed, in our case, the needle tract is quite difficult to visualise on follow-up MRI. Alternative ultrasound-guided methods such as the use of a balloon catheter to dilate a fenestration in the cyst wall may be considered in the future; however, the cost and availability of the needle technique may be particularly appealing in the developing world.

Conversely, we are aware of several risks and limitations relating to our technique—specifically the lack of direct visualisation and the proximity to the fornix, septal, and thalamostriate veins render the technique only possible for very large cysts presenting floridly through the foramen of Monro. Additionally, our technique should only achieve a fenestration of the cyst into the ventricular system, attempting to fenestrate into the prepontine cistern under ultrasound guidance would clearly pose a prohibitive risk of basilar arterial injury. The long-term outcomes of cysto-ventriculostomy relative to cysto-cisternostomy remain poorly studied; therefore, it is difficult to infer if this would be a weakness of an ultrasound-guided technique [2]. Finally, although in the contemporary era, many neurosurgeons are increasingly comfortable with ultrasound guidance for various techniques, lack of familiarity could certainly pose a barrier to uptake of this technique. Relevant to this last point, the closest description of anything approaching our technique within the existing literature is a historical description of fenestration suprasellar arachnoid cysts with the leukotome, which raised issues of the unfamiliarity/unreliability of radiological guidance in their series [7].

## Conclusion

We propose this relatively simple bedside technique to be considered a management option which may temporise, or perhaps definitively manage suprasellar arachnoid cysts in the neonatal period.

## Data Availability

No datasets were generated or analysed during the current study.
